# Functional characterization of full-length BARD1 strengthens its role as a tumor suppressor in neuroblastoma

**DOI:** 10.7150/jca.36164

**Published:** 2020-01-14

**Authors:** Flora Cimmino, Marianna Avitabile, Vito Alessandro Lasorsa, Lucia Pezone, Antonella Cardinale, Annalaura Montella, Sueva Cantalupo, Achille Iolascon, Mario Capasso

**Affiliations:** 1 Dipartimento di Medicina Molecolare e Biotecnologie Mediche, Università degli Studi di Napoli “Federico II”, Naples, Italy; 2 CEINGE Biotecnologie Avanzate, Naples, Italy; 3 IRCCS SDN, Naples, Italy

**Keywords:** Neuroblastoma, BARD1, tumor suppressor gene

## Abstract

*BARD1* is associated with the development of high-risk neuroblastoma patients. Particularly, the expression of full length (FL) isoform, FL *BARD1*, correlates to high-risk neuroblastoma development and its inhibition is sufficient to induce neuroblastoma cells towards a worst phenotype. Here we have investigated the mechanisms of FL *BARD1* in neuroblastoma cell lines depleted for FL *BARD1* expression. We have shown that FL *BARD1* expression protects the cells from spontaneous DNA damage and from damage accumulated after irradiation. We demonstrated a role for FL *BARD1* as tumor suppressor to prevent unscheduled mitotic entry of DNA damaged cells and to lead to death cells that have bypassed cell cycle checkpoints. FL *BARD1*-depleted cells that have survived to checkpoints acquire features of aggressiveness. Overall, our results show that FL *BARD1* may defend cells against cancer and prevent malignant transformation of cells.

## Introduction

Neuroblastoma is a pediatric malignancy that arises from the sympathetic nervous system. The cure of neuroblastoma patients remains a challenge for the pediatric oncologists; indeed, the number of long-term survivors of high-risk neuroblastoma with 5-year survival is 40%, despite decades of considerable international efforts to improve outcome 1. High-throughput sequencing-based studies have reported that recurrent mutations of single genes are infrequent in primary neuroblastoma with activating mutations in *ALK* and inactivating mutations in *ATRX*, and *TERT* rearrangements being the most frequent 2-4. Gain of function mutations in *ALK* in ~10% of cases has emerged as the only validated therapeutic target 4-6. Recent single-nucleotide polymorphism (SNP) based genome-wide association studies (GWAS) have identified several susceptibility neuroblastoma genes (*CASC15*, *BARD1*, *LMO1*, *DUSP12*, *HSD17B12*, *DDX4/IL31RA*, *HACE1*, *LIN28B*, *NEFL)* 7-12 and *BARD1* results to be the most strongly associated gene 10-13. Many of the identified loci impart oncogenic dependencies in established tumors.

*BARD1* is characterized by full length (FL) and diverse spliced isoforms. Several scientific evidences show that cancer-associated BARD1 isoforms antagonize the functions of FL BARD1 as tumor suppressor and act as a driving force for carcinogenesis. In particular, BARD1 oncogenic isoforms are often up-regulated and associated with negative prognosis in breast, ovarian, endometrial and lung cancers 14, 15. In particular, the isoform BARD1β is an oncogenic driver of high risk neuroblastoma tumorigenesis through interaction with Aurora family of kinases 16. Although FL BARD1 expression can have oncogenic effects 17-20, its role as tumor suppressor remains to be elucidated. Somatic acquired mutations of *BARD1* are relatively low frequent in cancer and, even if rare, *BARD1* mutations seem to drive malignant transformation 21, 22. Diverse *BARD1* SNPs with cis-effect on FL *BARD1* are identified as protective variants against high-risk neuroblastoma 10, 13, whereas variants with a cis-effect on isoform *BARD1β* are associated with high-risk neuroblastoma 23. Additionally, in our recent sequencing study, *BARD1* is enriched in rare, potentially pathogenic, germline variants 24.

The *BARD1* RING domain is an ubiquitin ligase forming a heterodimer with BRCA1, which also harbors a RING domain. The heterodimeric complex localizes at site of DNA damage and functions in the regulation of centrosome amplification and chromosome de-condensation 25, 26. Literature data report that *BARD1* and *BRCA1* gene knockouts have similar phenotypes demonstrating that both *BARD1* and *BRCA1* are essential for cell viability and maintenance of genome integrity 27, 28. Overall, both proteins may function individually, interacting with various proteins and the dissociation of the heterodimer might be regulated by posttranslational protein modifications such as phosphorylation, ubiquitination or parsylation. FL BARD1 protein not in complex with BRCA1 has emerged as key player in poly(ADP-ribose) (PAR) signaling after DNA damage 29 and its cytoplasmic localization is associated with pro-apoptotic activity 30, 31. Another BRCA1- independent function of BARD1 is observed at late stage of mitosis where FL BARD1 protein dissociates from BRCA1 and interacts with BRCA2 and Aurora kinase B, essential for the completion of cytokinesis 32.

We previously reported that the repression of FL *BARD1* is crucial for neuroblastoma cells proliferation and invasion 13. In this study, we further investigated FL *BARD1* in neuroblastoma cells to support the hypothesis of its role as tumor suppressor gene. We show that FL *BARD1* is involved in DNA damage response and FL *BARD1* depletion allows neuroblastoma cells to proceed in mitosis by avoiding cell cycle checkpoints. Based on these observations, we assume that accumulated mutations during DNA damage may not be repaired in absence of FL *BARD1* and thus, unrepaired cells might acquire features that are more aggressive. Additionally, we demonstrated a role for FL BARD1 as tumor suppressor that is independent of DNA damage response that needs major elucidation in the next future.

## 2. Material and Methods

### 2.1 Correlation analysis between the expression of FL BARD1 and all genes

Correlation analysis was performed through the R2 platform (r2.amc.nl) using the defaults parameters (FDR<0.01) and the above-mentioned dataset of 161 neuroblastoma tumors profiled by RNAseq through TARGET project. For this analysis, we used the transcript ENST00000260947 that identifies the FL *BARD1*. The Gene Ontology and KEGG pathway analyses were performed through the same R2 platform, on the genes that significantly correlated with FL *BARD1* expression.

### 2.2 Cell culture and Irradiation

The human SHSY5Y and SKNSH cell lines obtained from the American Type Culture Collection (respectively ATCC #CRL-2266 and #HTB-11) were grown in Dulbecco's Modified Eagle Medium (DMEM; Sigma) at 37 C, 5% CO2 in a humidified atmosphere. The medium was supplemented with 10% heat-inactivated FBS (Sigma), 1 mmol/L L-glutamine, penicillin (100 U/mL), and streptomycin (100mg/mL; Invitrogen). The cell lines were authenticated and early-passage cells were used for all the experiments. Irradiation (IR) treatment (160-kVp X-rays; 25mA; half-value layer of 0.3 mmCu) was administered using the RS2000 Biological Irradiator (RADSOURCE Technologies) at a dose rate of 5Gy (17.57 mGy /sec).

### 2.3 Production of Lentiviral particles and Infection of cell lines

To knockdown FL *BARD1* gene expression, pGIPZ Lentiviral shRNAmir targeting human *BARD1* were purchased from Open Biosystems (Thermo Fisher Scientific, Inc.). We used two different shRNA against FL *BARD1*: V2LHS_93186 and V3LHS_365581. A non-silencing pGIPZ Lentiviral shRNAmir was used as control (RHS4346). HEK293 were transfected with 10µg of shRNA plasmid DNA and 30µl of Trans-Lentiviral packaging Mix (OpenBiosystem) and 25µl TrasFectin (Bio-Rad) in 10mm plate. The supernatants (10 ml for points) were harvested after 24 hours, centrifuged at low speed to remove cell debris and filtered through a 0.45 μm filter 33, 34. In vitro transduction and determination of lentivector Titre was performer as already reported 35. After 48 hours of incubation, the transduced cells were examined microscopically for the presence of TurboGFP expression (70-90%). To obtain 100% GFP positive cells we added puromycin in the medium for additional 10 days.

### 2.4 Preparation of nuclear and cytosol extracts

Cells were suspended in cell lysis buffer [10 mM HEPES; pH 7.5, 10 mM KCl, 0.1 mM EDTA, 1 mM dithiothreitol (DTT), 0.5% Nonidet-40, 0.5 mM phenylmethylsulfonyl fluoride (PMSF), protease inhibitor cocktail (Sigma)] and incubated in ice for 15-20 min. Tubes are vortexed to disrupt cell membranes and then centrifuged at 12,000 g at 4°C for 10 min. The supernatant was stored at -80°C until further use as cytoplasmic extract. The pellets were washed thrice with cell lysis buffer and suspended in nuclear extraction buffer [20 mM HEPES (pH 7.5), 400 mM NaCl, 1 mM EDTA, 1 mM DTT, 1 mM PMSF] with protease inhibitor cocktail and incubated in ice for 30 min. Nuclear proteins were collected upon centrifugation at 12.000 rpm for 15 min at 4°C. Protein concentration was estimated by using Bradford's reagent (BioRad) [29].

### 2.5 Western blotting assay

Protein extracts were electrophoresed on polyacrylamide gel (Invitrogen) and transferred onto nitrocellulose membranes (Millipore). After 1 hour (h) blocking with 5% dry fat milk in p*hosphate*-buffered saline (PBS) containing 0.02% Tween-20, the membranes were incubated with the primary antibody overnight at 4 C° and with the secondary antibody for 1h at room temperature. Primary antibodies used are: anti-human BARD1 (cod-A300-263A, Bethyl, 1:1000), γH2AX (phosphoSer139) (cod-H5912, Sigma Aldrich, 1:1000), phosphor-p53(Ser-15) (cod-9284 Cell Signaling, 1:500), p53 (sc-6243, Santa Cruz, 1:500), Cyclin B (sc-752 Santa Cruz, 1:500), CDK1 (sc-54, Santa Cruz, 1:1000); phospho-H3 (06-570 Millipore). Mouse monoclonal anti-β-Actin antibody (cod-A5441, Sigma-Aldrich, 1:6000) and anti-H3 (cod-06-755, Millipore, 1:1000), were used as loading control for cytosol and nuclei extracts respectively. Secondary peroxidase-labeled antibody to rabbit IgG (cod041506, KPL) and to mouse IgG (cod041806, KPL) were diluted at 1:2000. Protein bands were visualized with enhanced chemiluminescence plus reagent (GE Healthcare). The protein bands image were acquired with GelDoc 2000 system (Bio-Rad) and the densitometry measurement was performed by Quantity One 4.5 tool (Bio-Rad).

### 2.6 Cell cycle distribution

Cells were seeded in cell culture 10-mm×20-mm dishes (Corning) at a density of 1×10^6^ cells. For the cell cycle analysis, 1×10^6^ cells were washed in PBS and suspended in 200 μl propidium iodide (50 μg/ml in PBS; Sigma), plus 50 μl RNaseA solution (100 μg/ml in water; Sigma) and NP40 (0/004% in PBS) and incubated at 37 °C for 3h in the dark. The cell-cycle distribution was analyzed by fluorescence-activated cell sorting (BD FACS, Canto II, BD Biosciences). The means (%) were calculated from two independent experiments.

### 2.7 Assay for caspase-3 activity

Caspase-3 activity was evaluated using Caspase Fluorescent (AFC) Substrate/Inhibitor QuantiPak (ENZO Life Sciences) following the manufacturer's protocol, and Microplate Imaging System (Bio-Rad) performed the measurement of enzymatic activity at 530nm. The means and standard deviations were calculated from two independent experiments.

### 2.8 Cell viability assay

Cells were grown for a total of 10 days after IR. Irradiated cells (IR) and not-irradiated cells (V) were seeded as six replicates into 96-well plates at a density of 10^4^ cells per well. After 7, 8, 9, and 10 days of IR, the metabolic activities of the samples were assessed as a surrogate marker for cell proliferation, using the 3-(4, 5-dimethylthiazol-2-yl), 5-diphenyltetrazolium bromide assay, according to the manufacture protocol (Promega). The means and standard deviations were calculated from two independent experiments.

### 2.9 Colony formation assay in soft agar

Two hundred thousand cells were plated in 0.35% agar on a bottom layer of 1% agar in 35-mm dishes (Corning). The plates incubated at 37 °C for 4 weeks were stained with 0.01% crystal violet. Colonies with 20 cells or more were counted. The means and standard deviations were calculated from three independent experiments.

## 3. Results

### 3.1 FL BARD1 functions in DNA damage response

We have previously evaluated the mRNA levels of FL *BARD1* in high-risk neuroblastomas compared with low and intermediate risk neuroblastomas and in patients with favorable neuroblastomas (Stage 4s) compared to metastatic neuroblastomas (Stage 4) 36. The results showed that patients with high-risk and metastatic tumors have reduced FL *BARD1* expression. These findings have encouraged us to further investigate the biological role of FL *BARD1* as tumor suppressor in neuroblastoma.

**Figure 1:**
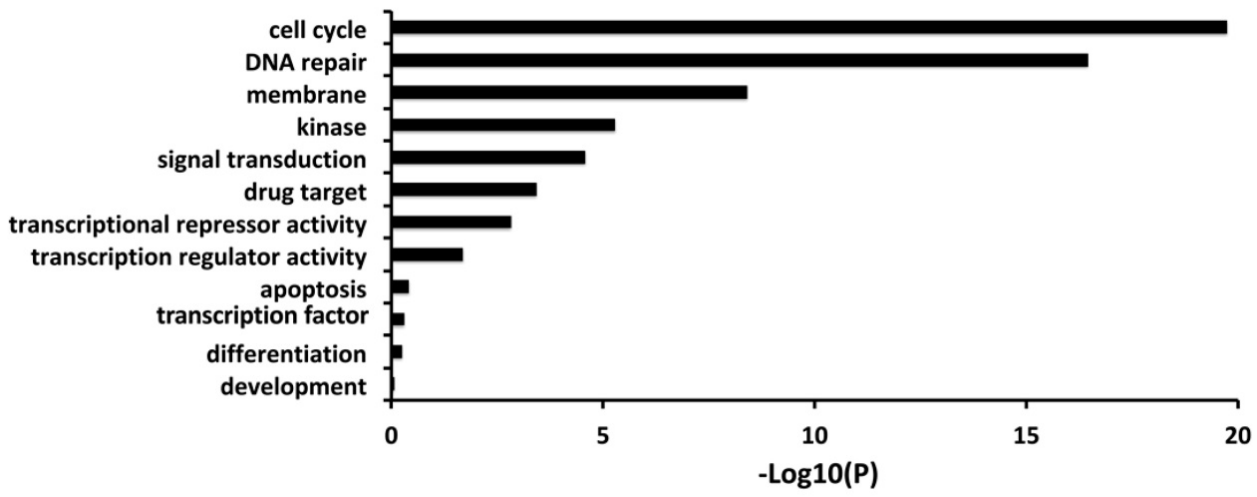
**FL *BARD1* expression correlates with expression of genes involved in the cell cycle and DNA repair**. Gene Ontology results for genes that significantly correlated with FL *BARD1* expression (RNAseq) in 161 neuroblastoma tumors. P-value is reported as -Log10 (P) on X-axis.

Firstly, we carried out a correlation analysis between FL *BARD1* expression *versus* all genes in 161 neuroblastomas profiled by RNAseq that allows distinguishing among alternative spliced transcripts. The gene ontology and pathways analysis showed that the expression of FL *BARD1* (ENST00000260947) is correlated with the expression of genes involved in cell cycle and DNA repair (**Figure 1, Table S1).**

**Figure 2:**
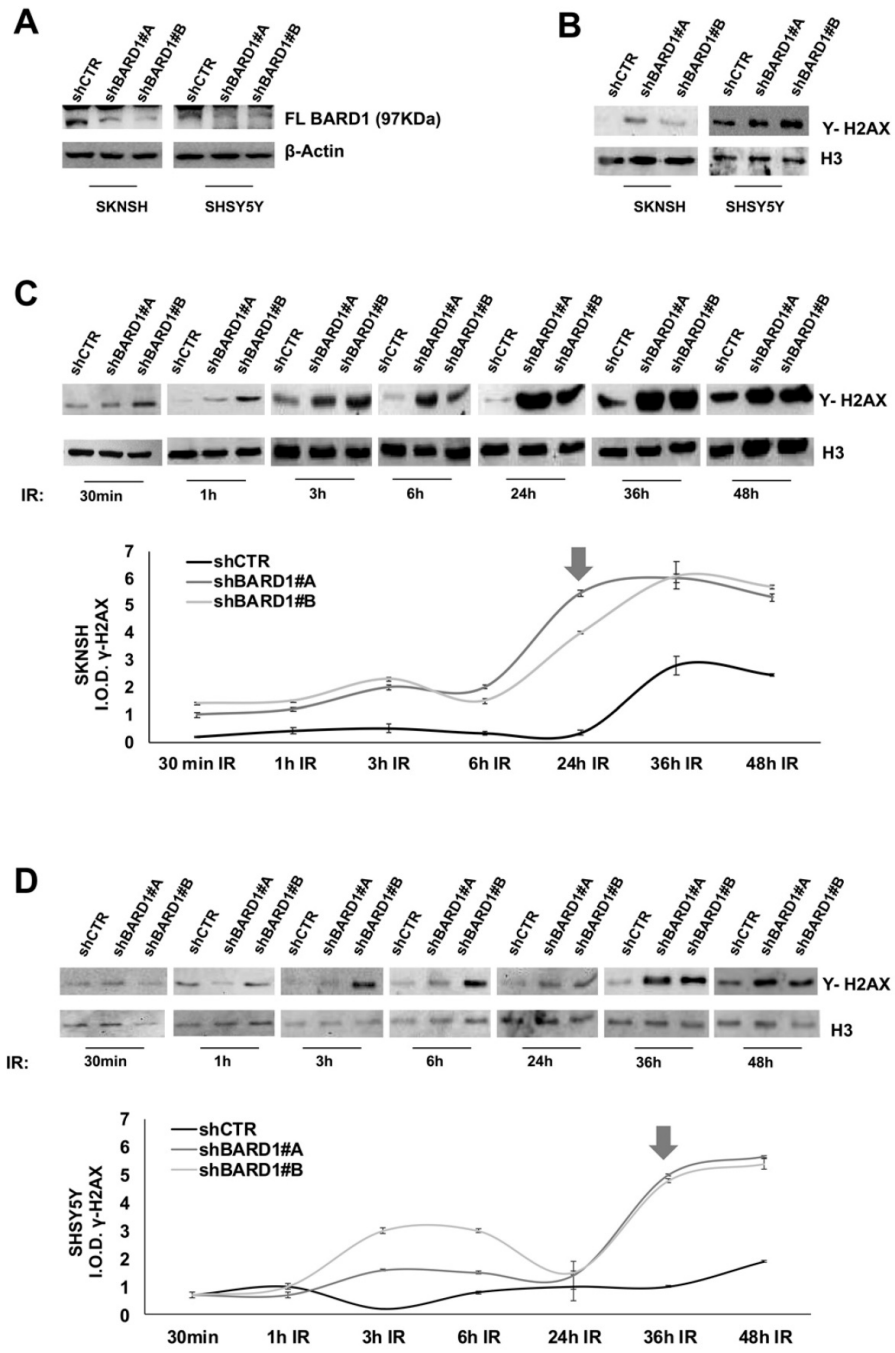
** FL *BARD1* functions in DNA damage response.** SKNSH and SHSY5Y cell lines were silenced for FL *BARD*1 expression upon transfection with lentiviral plasmids (shBARD1#A, shBARD1#B). Unsilenced control cells were transfected with plasmid shCTR. The efficiency of short harpin silencing was verified by western blotting, using an antibody against FL BARD1 isoform. The molecular weight of FL BARD1 isoform is reported. The higher band in the blot is an aspecific staining. β-Actin levels were used as loading control (A). The detection of Υ -H2AX protein was verified in nuclear extract of silenced (shBARD1) and unsilenced control (shCTR) cells, by western blotting. Antibody against histone H3 was used as loading control (B). SKNSH shBARD1 and shCTR cells (C) and SHSY5Y shBARD1 and shCTR cells (D) were treated with 5 Gy IR. The expression of Υ- H2AX was measured by western blotting in a time-course (30 min, 1h, 3h, 6h, 12h, 24h, 36h, 48h) after IR. The integral optical density (IOD) of Υ- H2AX protein bands were measured and normalized respect to loading control protein band H3. The arrows indicate the higher increment of Υ- H2AX in each cell line. The experiments were repeated twice.

To investigate a role for FL *BARD1* as tumor suppressor, two neuroblastoma cell lines (SKNSH and SHSY5Y) were depleted for FL *BARD1* expression upon stable transfection with lentiviral plasmids expressing short hairpin RNA against *BARD1* (shBARD1#A, ahBARD1#B). Unsilenced cells were transfected with control plasmid (shCTR). In both cell lines the efficient depletion of FL *BARD1* in shBARD1#A and shBARD1#B transfected cells (shBARD1#A and shBARD1#B cells) in contrast with shCTR transfected cells (shCTR cells) was verified by western blotting (**Figure 2A**). Since *BARD1*β expression might establish competing mechanisms, we verified the absence of *BARD1*β increment in FL *BARD1*-depleted cells (**Figure S1**). To confirm the specificity of our findings in neuroblastoma cells, all below described experiments have been replicated in two additional cell lines, shown in supplementary data.

In absence of induced DNA damage, we observed an increment of γH2AX protein in shBARD1#A and shBARD1#B cells compared to shCTR cells (**Figure 2B**). To understand the potential mechanism of FL *BARD1* in DNA damage, we treated SHSY5Y and SKNSH cells (shBARD1#A, shBARD1#B, shCTR) with 5Gy X-ray to induce DNA damage and evaluated γH2AX protein increment after different time points from irradiation (IR) exposure (30 min, 1h, 3h, 6h, 24h, 36h, 48h). To visualize the γH2AX protein expression levels, the protein bands intensities were measured by densitometry and normalized with respect to loading control H3, as shown in the graphs (**Figure 2C-D**). The analysis of γH2AX expression showed higher levels of γH2AX in shBARD1#A and shBARD1#B cells than shCTR cells, with strong increment after 24h in SKNSH shBARD1 cells (**Figure 2C**) and after 36h in SHSY5Y shBARD1 cells (**Figure 2D**). These findings show that higher levels of FL *BARD1* expression might protect neuroblastoma cells from spontaneous damages and from damages accumulation after IR.

### 3.2 FL BARD1 functions in regulating G2/M cell cycle phase and apoptosis

**Figure 3:**
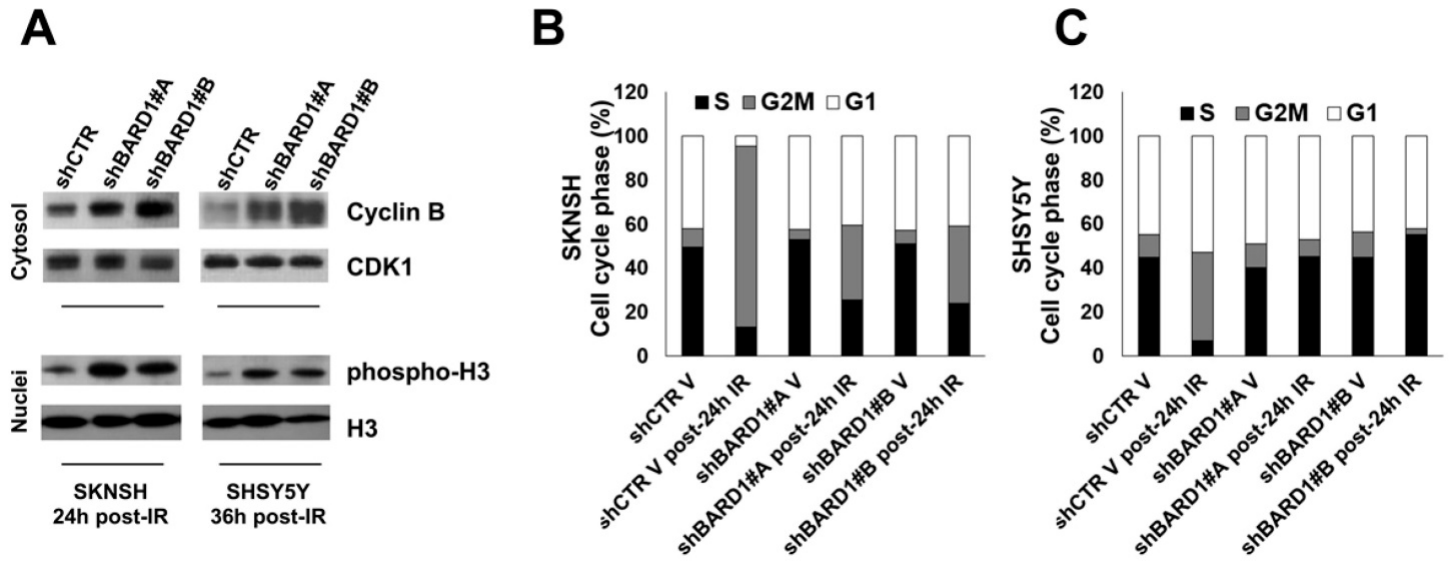
** FL BARD1 functions in G2-M cell cycle phase.** Cyclin B and CDK1 protein levels were verified in cytosol extracts and phospho-H3 and H3 protein levels were verified in nuclei extracts by western blotting in SKNSH (24 hours post-IR) and in SHSY5Y (36 hours post-IR) cells (A). Cell cycle distribution phases were reported as mean percentages between two experiments, in SKNSH shBARD1 and shCTR IR cells and non-irradiated cells (V) (B) and in SHSY5Y shBARD1 and shCTR IR cells and non-irradiated cells (V) (C).

The G2-M DNA damage checkpoint ensures that cells do not initiate mitosis before they have a chance to repair damaged DNA after replication. The transition of cells from the G2 phase to the M phase is driven by critical cell cycle proteins, cyclin B and Cdc25C, which were poly-ubiquitinated and degraded by FL BARD1 in complex with BRCA1 37, 38. Here, to evaluate the essential role of FL BARD1 for G2-M checkpoint activation we chose post-IR time points where we have previously observed the higher γH2AX protein increment in FL *BARD1*-depleted cells (**Figure 2C-D**). In line with literature data, in FL *BARD1*-depleted cells (shBARD1#A and shBARD1#B) the levels of cyclin B are higher than in unsilenced cells (shCTR) (**Figure 3A**). Furthermore, the degradation of cyclin B in post-IR SKNSH and SHSY5Y shCTR cells goes with an increase of cells accumulated in G2 phase of the cell cycle, compared to not-irradiated SKNSH and SHSY5Y shCTR cells (V) (**Figure 3B-C**). In SKNSH cell line, the increase of post-IR shBARD1 cells accumulated in G2 phase compared to non-irradiated shBARD1 (V) cells was less than that observed in post-IR shCTR cells compared to non-irradiated shCTR (V) cells (**Figure 3B**). Conversely in SHSY5Y cell line, we do not observe an increase of shBARD1 cells accumulated in G2 phase, compared to shBARD1 non-irradiated cells (V) (**Figure 3C**). These results suggest that cells depleted for FL *BARD1* expression have a defective G2-M checkpoint and enter mitosis before repairing their DNA. Increase of phopsho-H3 levels in post-IR shBARD1 cells respect to shCTR cells further confirmed these observations (**Figure 3A**).

**Figure 4:**
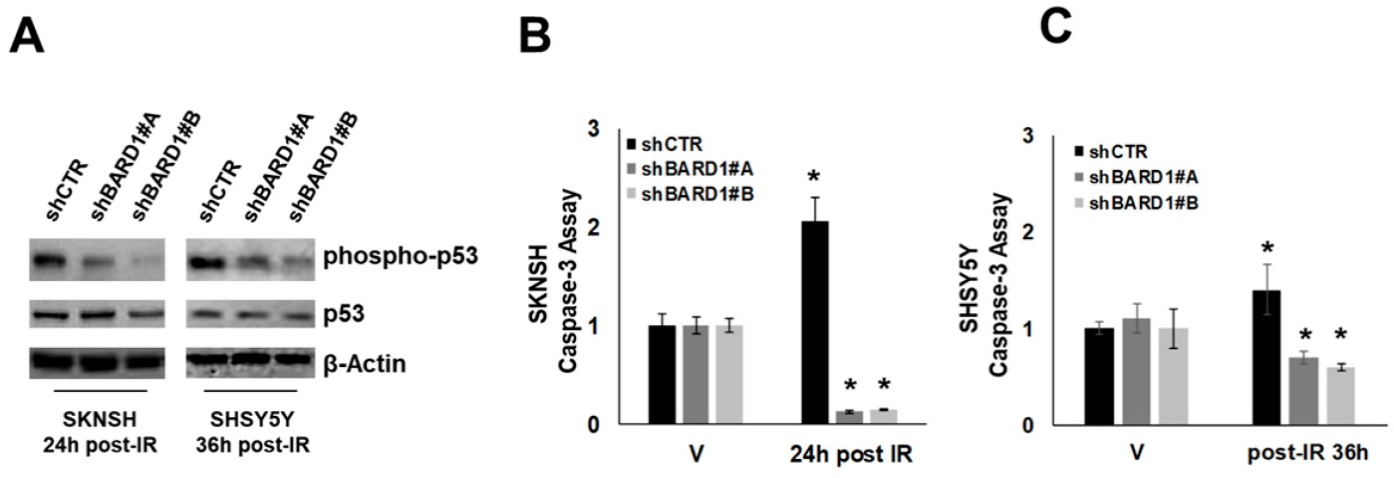
** FL BARD1 functions in regulating apoptosis.** Phospho-p53 and p53 and β-Actin protein levels were verified by western blotting in shBARD1 and shCTR cells, in SKNSH (24 hours post-IR) and SHSY5Y (36 hours post-IR) cell lines (A). Caspase-3 activity was evaluated in SKNSH shBARD1 and shCTR IR and V cells (B) and in SHSY5Y shBARD1 and shCTR IR and V cells (C). The asterisk is indicative of p-value ≤ 0.05. The experiments were repeated twice.

Literature data report that FL BARD1 not in complex with BRCA1 acts as an adaptor for p53, enabling it to be targeted for ATM/ATR-directed serine-15 phosphorylation (p53Ser-15) following IR/ UV-induced DNA damage in several cell types. This phosphorylation is required for p53 apoptotic function 25, 26. We observed that the depletion of FL *BARD1* (in shBARD1 cells) disrupted p53Ser-15 in post-IR SKNSH and SHSY5Y cells whereas p53Ser-15 was observed in post-IR SKNSH and SHSY5Y shCTR cells (**Figure 4A**). The impaired p53 function is further confirmed by the decrease in caspase-3 activity in post-IR shBARD1 cells compared to post-IR shCTR cells, both in SKNSH and SHSY5Y cell lines. Caspase activity in post-IR cells was represented as a ratio to caspase activity in non-irradiated (V) cells (**Figure 4B-C**). These observations suggest a role for FL *BARD1* to drive cells towards a protective arrest into apoptosis after initial DNA damage. SKNSH and SHSY5Y cells are both p53-wild type. To strength FL BARD1 involvement in DNA damage response, we verified FL BARD1 involvement in G1 and G2 checkpoints in two additional cell lines p53-mutated: SKNAS cells show homozygous deletion of exons 10-11 of p53 39 and SKNFI cells show missense mutation located in exon 7 of p53 40. As reported in the supplementary data, in shCTR and shBARD1 p53 mutated cells there is a basal level of p53 phosphorylation at DNA damage, which indicates the lack of p53 activation and G1 checkpoint. On the contrary, we observed cyclin B degradation and the increase of phospho-H3 levels in shBARD1 cells compared to shCTR cells, which indicates the FL BARD1 control of the G2 checkpoint (**Figure S2-S3**).

### 3.3 Loss of FL BARD1 promotes cells proliferation and cells growth and increases cells clonogenic activity

**Figure 5:**
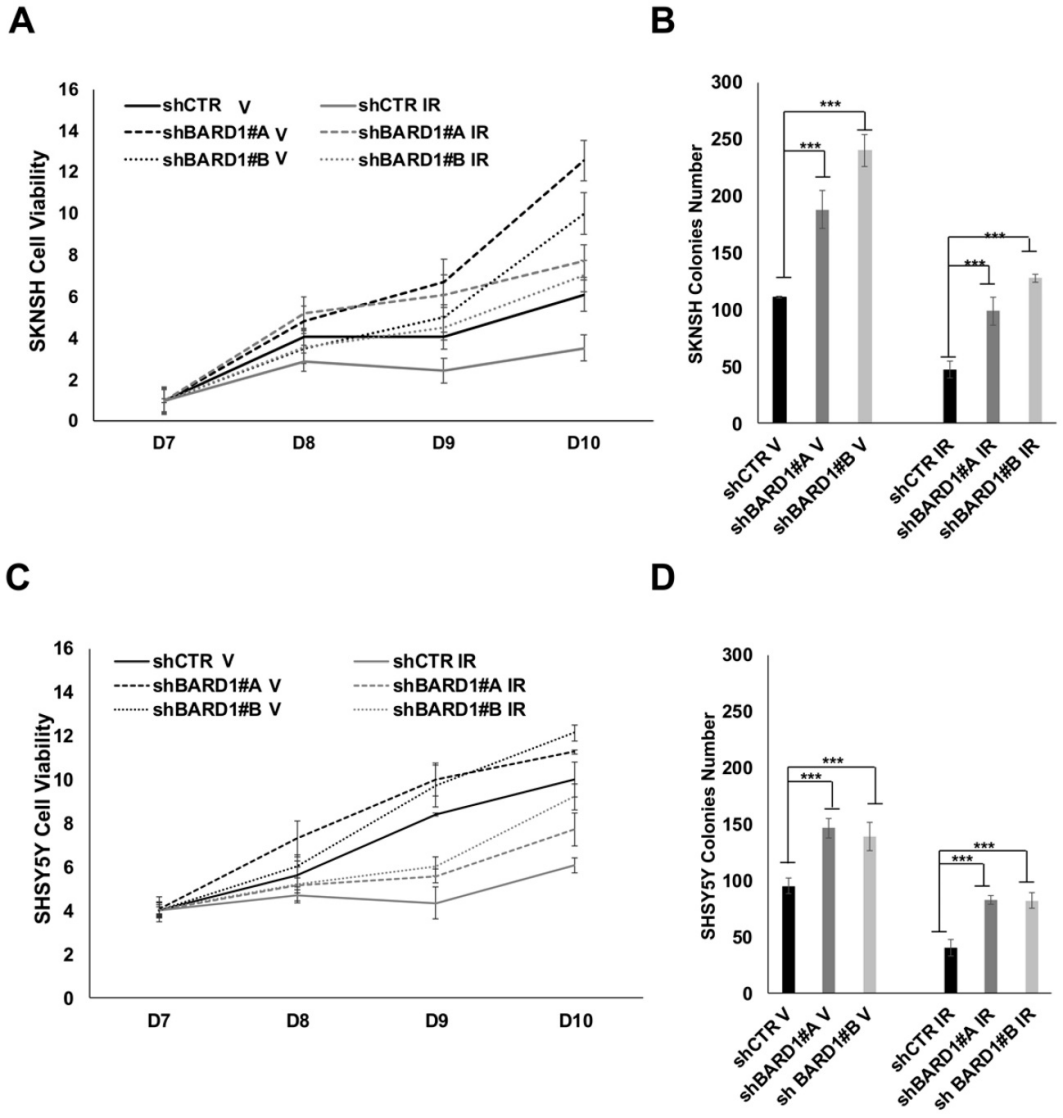
** FL *BARD1* depletion promotes cells proliferation and cells growth.** Cell proliferation assay was performed after seven days from IR (D7). SKNSH shBARD1 (V and IR) and shCTR (V and IR) cells viability were evaluated in the following 8 (8D), 9 (9D) and 10 days (10D) from IR and normalized respect to D7 (A). Soft agar assay was performed for the same cells and colonies number for each experimental point is reported on Y-axis (B). SHSY5Y shBARD1 (V and IR) and shCTR (V and IR) cells viability were evaluated in the following 8 (8D), 9 (9D) and 10 days (10D) from IR and normalized respect to D7 as shown in (C). Soft agar assay was performed for the same cells and colonies number for each experimental point in reported on Y-axis (D). The asterisks show the increments of colonies number with P<0.05 in shBARD1 V cells compared to shCTR V cells and in shBARD1 IR cells compared to shCTR IR cells (B, D). Cell viability assays were repeated twice, colony formation assays were repeated three times.

We evaluated cells proliferation and cells growth ability in soft agar of SKNSH, SHSY5Y, SKNAS and SKNFI cells depleted or not of FL *BARD1* expression. In both proliferation and soft agar assays, shBARD1 V cells are more proliferating and growing than shCTR V cells, showing a role of FL BARD1 tumor suppressor independent from induced DNA damage (**Figure 5; Figure S4**), as previously shown 13.

We demonstrated that FL *BARD1* depletion influences clonogenic activity in post-DNA damaged neuroblastoma cells. Cells proliferation of post-IR shCTR and shBARD1 cells was evaluated seven days after IR (D7) and in the following eight (D8), nine (D9) and ten (D10) days post-IR. Interesting to note, shBARD1 IR cells show higher cell viability (*P* < 0.05, D9, D10 SKNSH; *P* < 0.05 D9, D10 SHSY5Y; **Figure 5A, C**) and higher colony numbers in soft agar assay than shCTR IR cells (*P* < 0.05, **Figure 5B, D**). Loss of FL *BARD1* increasing clonogenic activity was confirmed in two additional cell lines, SKNAS and SKNFI, as shown in the supplementary data (**Figure S4**).

Overall, proliferation rate and growth ability in soft agar decrease in post-IR cells respect to V cells (both shCTR and shBARD1 cells) except in SKNAS cells, probably because intrinsic irradiation-sensitivity differs among cell lines.

## 4. Discussion

BARD1 and BRCA1 form a heterodimer via their N-terminal RING finger domains. This interaction is essential for BRCA1 stability and for relocation of BRCA1 to DNA damage sites. BRCA1/BARD1 heterodimer acts as an E3 ubiquitin ligase that ubiquitinates RNA polymerase II, preventing the transcription of the damaged DNA, and restoring genetic stability. Although BARD1 function is associated with the function of heterodimer, on the other hand, BARD1 also acts independently of BRCA1. Indeed, BARD1 expression, upregulated by genotoxic stress, is involved in apoptosis through binding and stabilizing p53 independently of BRCA1. Furthermore, FL BARD1 may interact with additional partners through its protein domains and act in several pathways essential for cells vitality but these aspects need more elucidations 22.

*BARD1* locus is one of the most significant and robustly replicated association signals enriched in high-risk subset of neuroblastoma 10, 13. We found high-risk variants that fall into promoter correlate with low expression of FL *BARD1* and with neuroblastoma development. On the other hand, high-risk variants that fall into introns correlate with high expression of cancer-associated *BARD1*β isoform that antagonizes FL *BARD1* functions and acts as a driving force for carcinogenesis. Although we have previously shown that down-regulation of FL *BARD1* has oncogenic effects 13, a role for FL *BARD1* as tumor suppressor gene has not been examined in neuroblastoma cells.

In the present study, we show that FL *BARD1* expression correlates with the expression of genes involved in DNA repair and cell cycle in neuroblastoma samples, probably due to BARD1/BRCA1 heterodimer function. From literature, cells deficient of BRCA1 tend to accumulate DNA damage by increasing γH2AX phosphorylation that can further lead to genome instability and carcinogenesis 41. In the same way, our data show that neuroblastoma cells deficient in FL *BARD1* tend to accumulate γH2AX phosphorylation spontaneously or upon DNA insults suggesting higher levels of FL *BARD1* expression protect neuroblastoma cells from DNA damages accumulation while lower levels of FL *BARD1* make cells prone to carcinogenesis by accumulating more mutations.

G1 and G2/M checkpoints are important steps to avoid that cancer cells treated with DNA damage agents could be able to repair the damage and continue to proliferate accumulating more damages than before. Literature reports that BRCA1 targets G2/M cell cycle proteins for degradation 38. Here we show that FL BARD1, as part of the heterodimer BRCA1/BARD1, prevent unscheduled mitotic entry of DNA damaged neuroblastoma cells via a mechanism requiring downregulation of cyclin B/Cdk1 and cell cycle arrest at the G2-M boundary. Indeed cells depleted of FL *BARD1* have a defective G2-M checkpoint and enter mitosis before repairing their DNA. After initial DNA insults, we observe a p53 inactivation and a decrease in apoptosis in FL *BARD1*-depleted cells. This is in accordance with literature data showing that BARD1 acts as an adaptor for p53, enabling it to be targeted for ATM/ATR-directed phosphorylation following IR/ UV-induced DNA damage 31. These data suggest that FL BARD1 through p53Ser-15 further drives DNA damaged cells towards a protective arrest into apoptosis. The control of *FL* BARD1 on apoptosis through p53 stability fails in neuroblastoma p53-mutated cells but the control of *FL BARD1* on p53-independent G2 cell cycle checkpoint remains. Taken together, our data clarify that higher expression of FL BARD1 is necessary to arrest cells in G1 and G2/M checkpoints following IR and FL BARD1 is still necessary to arrest cells in G2/M checkpoint in p53-mutated cells.

Increased clonogenic activities in post-DNA damage cells further shows the role of tumor suppressor for FL *BARD1* in DNA damage. The increase in cell proliferation and cell growth in soft agar in neuroblastoma cells depleted of FL *BARD1* expression shows an additional role of tumor suppressor not dependent on DNA induced damage, according to our previous report 13. These findings suggest that higher FL *BARD1* expression in primary neuroblastoma is a protective factor to defend cells against spontaneous DNA insults and thus preventing cells malignant transformation. In the present study, we have not investigated if that tumor suppressor role for FL BARD1 is dependent from BRCA1, but we should consider that FL BARD1 might act in additional pathways involved in carcinogenesis through additional binding partners that remain not investigated.

Neuroblastoma derived cell lines with genomic alterations of DNA-damage response associated genes and with *BRCA1* or 2 and *BARD1* mutations exhibited sensitivity to PARP1 inhibitors (PARP1i) 42. Particularly, neuroblastoma patients with 11q-loss (with ATM haploinsufficiency) define a subgroup of patients with higher sensitivity to PARP1i 43. In these cells deficient of homologous recombination repair, PARP1i lock PARP1 onto DNA, blocking progression of a replication fork and leading cells to synthetic lethal death 44, 45. Since FL BARD1 acts in heterodimer with BRCA1 in DNA double-strand-break repair and has shown to bind PARP1 in DNA damage response 29, it is reasonable to assume that FL *BARD1* deficient cells could less efficiently repair the double strand breaks generated by PARP1i and die quickly.

The presented data support the onco-suppressor role of FL BARD1 in neuroblastoma and its involvement in DNA repair and cell cycle and provides evidence that abnormal expression or genetic mutations of *BARD1* might be a reliable biomarker for tumor prevention opening the way to new approach for therapy decision making. Nevertheless, FL BARD1 characterization is incomplete in cancer and major elucidation, related to mechanisms by which FL BARD1 results in potential oncogenic vulnerabilities, needs in the next years.

## Supplementary Material

Supplementary materials and methods, figures.
