# Supplementary material for: Functional characterization of full-length BARD1 strengthens its role as a tumor suppressor in neuroblastoma
Source: J Cancer. 2020 Jan 14;11(6):1495–504. doi: 10.7150/jca.36164 (PMC6995383; doi:10.7150/jca.36164)
Supplement: Supplementary file 1 — Supplementary materials and methods, figures. [file jcav11p1495s1.pdf]

Supplementary Material

BARD1 silencing affects FL *BARD1* and *BARD1*β expressions.

Literature reports BARD1β is a dominant negative of FL BARD1 in cancer cells. We verified that shRNA silencing of BARD1 affects the expression of FL *BARD1* and of *BARD1*β in the same cells, thus avoiding the establishment of competing mechanisms. *BARD1*β is characterized by deletion of exons 2 and 3 (Cancer Res. 2007 Dec 15;67(24):11876-85.); to assess its specific mRNA expression we used primers targeting exon 1/4 boundary. We verified that basal levels of *BARD1*β expression in all four neuroblastoma cell lines (SHSY5Y, SKNSH, SKNAS, SKNFI) is very low compared to FL *BARD1* expression levels (**Figure S1-A**). The silencing efficacy on both FL *BARD1* and *BARD1*β expressions was verified by RT-PCR. The results are represented in percentage, where the expression of the two isoforms in shCTR was considered 100%. A shown, both FL BARD1 and *BARD1*β expressions decrease upon shBARD1 silencing (**Figure S1-B**).

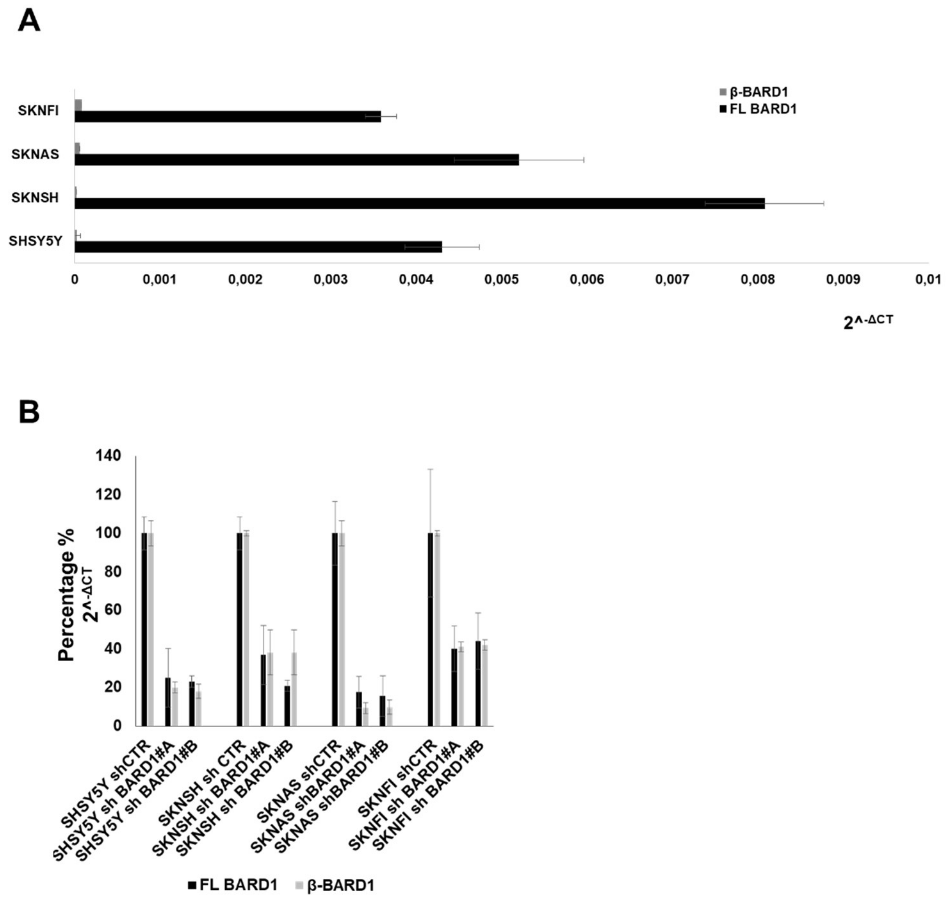

Figure S1. BARD1 silencing effect on FL BARD1 and *BARD1*β expression. The expression levels of both FL *BARD1* and BARD1β isoforms was evaluated by RT-PCR. In (A) is shown the basal levels of both isoforms in all four neuroblastoma cell lines. X-Axis show $2^{\wedge-\Delta CT}$ values. In (B) the expression of both isoforms was evaluated in shCTR and shBARD1 cells. The $2^{\wedge-\Delta CT}$ values are reported in percentage where $2^{\wedge-\Delta CT}$ values in shCTR cells was 100% for both FL *BARD1* and *BARD1*β expressions.

FL BARD1 functions in DNA damage response

We verified FL BARD1 involvement in G1 and G2 checkpoints in two additional neuroblastoma cell lines, both p53-mutated: SKNAS cells show homozygous deletion of exons 10-11 of p53 (Biochem Biophys Res Commun. 2007 Mar 23;354(4):892-8) and SKNFI cells show missense mutation located in exon 7 of p53 (Mol Cancer Ther. 2011 Jun;10(6):983-93). SKNAS and SKNFI cells were depleted for FL *BARD1* expression upon stable transfection with lentiviral plasmids expressing short hairpin RNA against *BARD1* (shBARD1#A, ahBARD1#B). Unsilenced cells were transfected with control plasmid (shCTR). In both cell lines the efficient depletion of FL *BARD1* in shBARD1#A and shBARD1#B transfected cells (shBARD1#A and shBARD1#B cells) in contrast with shCTR transfected cells (shCTR cells) was verified by western blotting (**Figure S2-A**). In absence of induced DNA damage, we observed an increment of γH2AX protein in shBARD1#A and shBARD1#B cells compared to shCTR cells (**Figure S2-B**). To understand the potential mechanism of FL BARD1 in DNA damage, we treated SKNAS and SKNFI cells (shBARD1#A, shBARD1#B, shCTR) with with 10Gy X-ray and 20Gy X-ray respectively to induce DNA damage and evaluated γH2AX protein increment after 48 hours from irradiation (IR) exposure. The western blot showed higher levels of γH2AX in shBARD1#A and shBARD1#B cells than shCTR cells, in both cell lines (**Figure S2-C**). Overall, these findings highlight that higher levels of FL *BARD1* expression might protect neuroblastoma p53-mutated cells from spontaneous damages and from damages accumulation after IR.

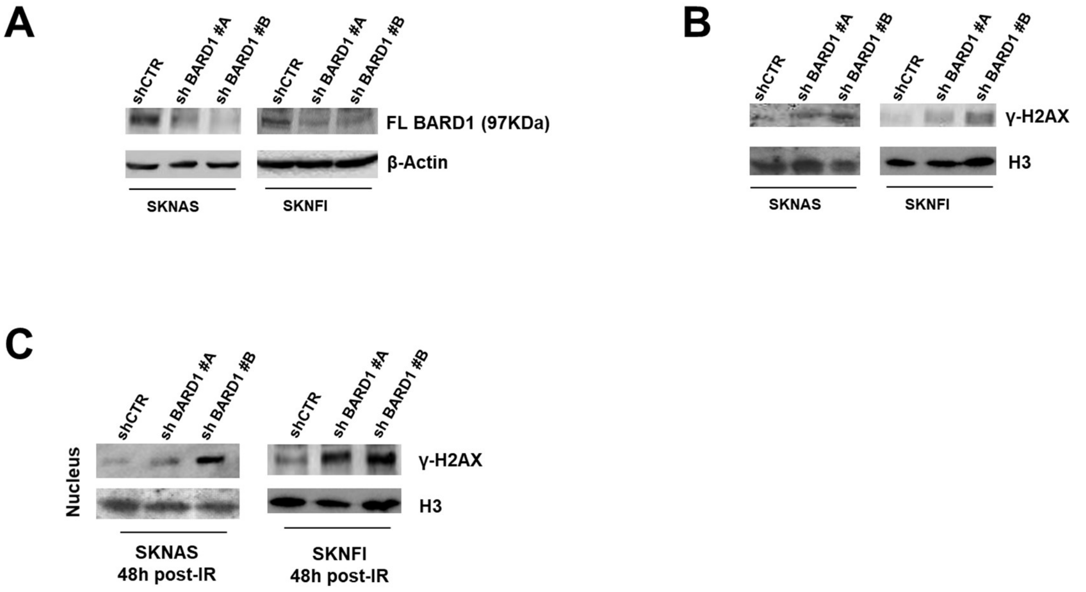

Figure S2. FL *BARD1* functions in DNA damage response. SKNAS and SKNFI cell lines were silenced for FL *BARD1* expression upon transfection with lentiviral plasmids (shBARD1#A, shBARD1#B). Unsilenced control cells were transfected with plasmid shCTR. The efficiency of short harpin silencing was verified by western blotting, using an antibody against FL BARD1 isoform. The molecular weight of FL BARD1 isoform is reported. Antibody against β-Actin was used as loading controls (A). The detection of γ-H2AX protein was verified in nuclear extract of silenced (shBARD1) and unsilenced control (shCTR) cells, by western blotting. Antibody against histone H3 was used as loading controls (B). SKNAS shBARD1 and shCTR were treated with 10 Gy IR. The expression of ϒ- H2AX was measured by western blotting at 48h after IR (C). SKNFI shBARD1 and shCTR were treated with 20 Gy IR. The expression of ϒ- H2AX was measured by western blotting at 48h after IR (D).

FL BARD1 functions in regulating G2/M phase in Neuroblastoma cells p53-mutated

We evaluated the essential role of FL BARD1 for G2-M checkpoint activation in SKNAS and SKNFI damaged cells at 48 hours post-IR time point where we have previously observed γH2AX protein increment in FL *BARD1* depleted cells. In line with data obtained in SKNSH and SHSY5Y cell lines, in cells FL *BARD1* depleted (shBARD1#A and shBARD1#B) the levels of cyclin B are higher than in unsilenced cells (shCTR). These results further confirm that cells depleted for FL *BARD1* expression have a defective G2-M checkpoint and enter mitosis before repairing their DNA. The increment of the levels of phopsho-H3 in post-IR FL *BARD1* silenced cells (shBARD1) respect to unsilenced cells (shCTR) further confirm these observations. We observed a baseline p53-phosphorylation in both shCTR and shBARD1 cells, indicative of lack of p53 activation upon DNA damage and of lack of G1 checkpoint in p53-mutated cells. Overall, these observations highlight FL BARD1 role in G2 checkpoints and lack of FL BARD1 control of G1 checkpoint in DNA damaged p53-mutated cells (**Figure S3)**.

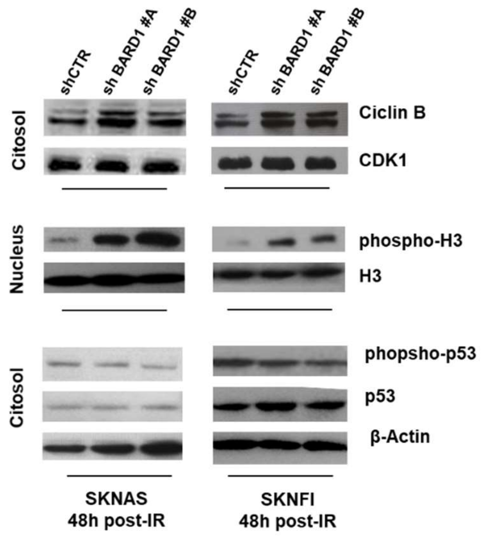

Figure S3. FL *BARD1* functions in G2-M but not in G1 cell cycle checkpoints in p53-mutated cells. At 48 hours post-IR, the expression of Cyclin B and CDK1 was verified in cytosol extracts and the expression of phospho-H3 and H3 was verified in nuclei extracts by western blotting in shBARD1 and shCTR cells (A). At 48 hours post-IR, the expression of phospho-p53 and p53 and β-Actin were verified by western blotting in cytosol extracts of both shBARD1 and shCTR cells.

Loss of FL BARD1 promotes cells proliferation and cells growth and increases cells clonogenic activity in neuroblastoma p53-mutated cells

To strength FL BARD1 role as tumor suppressor, we evaluated cells proliferation and cells growth ability in soft agar of cells depleted of FL *BARD1* and cells not depleted, in two additional neuroblastoma cell lines, both p53-mutated.

In this study we evaluated that shBARD1 V cells are more proliferating (cell viability assays) and growing (soft agar assays) than shCTR V cells, highlighting a role of FL BARD1 tumor suppressor independent from induced DNA damage (**Figure S4-A, B**).

In this study, we have further verified that FL *BARD1* depletion increases clonogenic activity in post-DNA damaged neuroblastoma cells. We evaluated the proliferation of post-IR shCTR and shBARD1 cells, particularly seven days after IR (D7) and in the following eight (D8), nine (D9) and ten (D10) days from IR, and we evaluated post-IR shCTR and shBARD1 cells growth ability in soft agar. Interesting to note, shBARD1 IR cells show higher cell viability and higher colony numbers in soft agar assay than shCTR IR cells ($P < 0.05$, **Figure S4-A, B**). Overall, proliferation rate and growth ability in soft agar decreased in SKNFI post-IR cells respect to V cells (both shCTR and shBARD1) but not in SKNAS cells, because intrinsic irradiation-sensitivity differs between cell lines

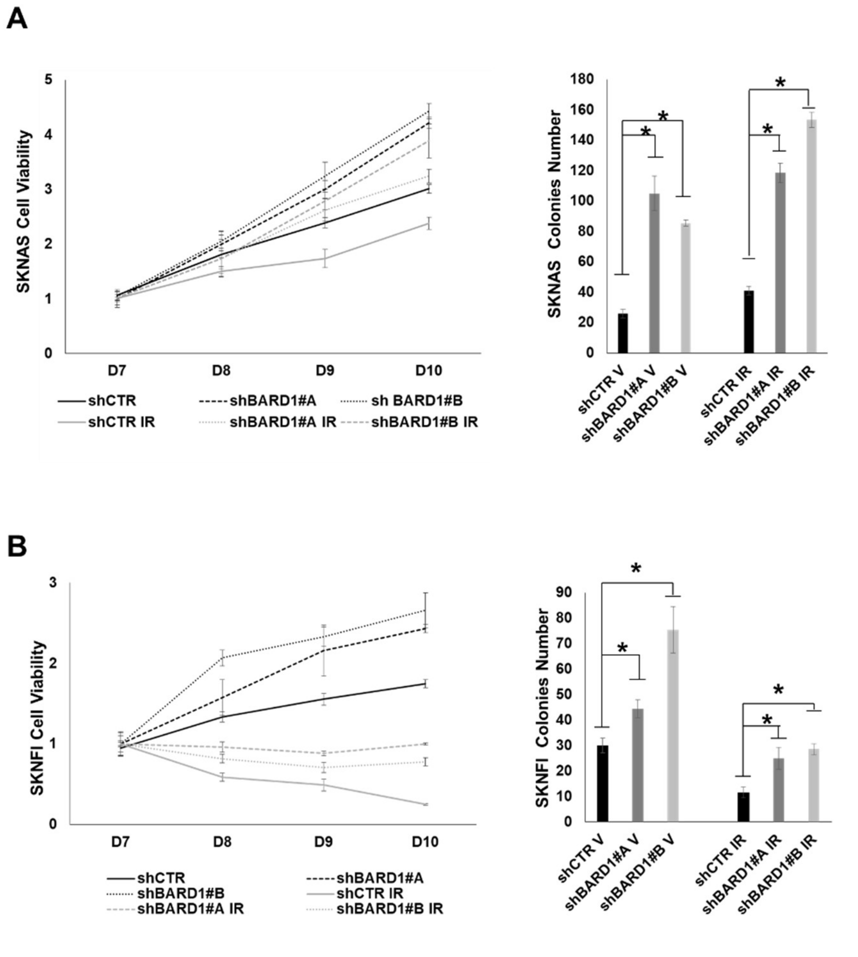

Figure S4. FL *BARD1* depletion promotes cells proliferation and cells growth in neuroblastoma p53-mutated cells. Cell proliferation assay was performed after seven days from IR (D7). shBARD1 and shCTR V and IR cells viability were evaluated in the following 8 (8D), 9 (9D) and 10 days (10D) from IR and normalized respect to D7 (A-B). Soft

agar assay was performed for the same cells and colonies number for each experimental point in reported on Y-axis (A-B). The asterisks show the increments of colonies number with $P<0.05$ in shBARD1 V cells compared to shCTR V cells and in shBARD1 IR cells compared to shCTR IR cells.

Supplementary Material and Methods

Cell culture and Irradiation

The human SKNAS and SKNFI cell lines obtained from the American Type Culture Collection (respectively ATCC #CRL-2137 and #CRL2142) were grown in Dulbecco's Modified Eagle Medium (DMEM; Sigma) at 37 C, 5% CO_2 in a humidified atmosphere. The medium was supplemented with 10% heat-inactivated FBS (Sigma), 1 mmol/L L-glutamine, penicillin (100 U/mL), and streptomycin (100mg/mL; Invitrogen). The cell lines were authenticated and early-passage cells were used for all the experiments. Irradiation (IR) treatment (160-kV X-rays; 25mA; half-value layer of 0.3 mm Cu) was administered using the RS2000 Biological Irradiator (RadSource Technologies) at a dose rate of 10Gy (SKNAS) and 20Gy (SKNFI) (17.57 mGy /sec).

Real Time-PCR

The mRNA expression levels of FL *BARD1* and *BARD1*β were analyzed using quantitative Real-time PCR. Total RNA extraction using TRIzol LS Reagent (Invitrogen) and cDNA retrotranscription using the SensiFAST cDNA Synthesis Kit (Bioline) were performed according to the manufacturer protocol. The cDNA samples were diluted to 20 ng/μl. Gene-specific primers were designed by using Primer Express 3.0 (Applied Biosystems, ABI). Primers to assess *BARD1*β expression target the exon 1/4 boundary (PMID: 22350409) and primers to assess the FL *BARD1* expression target exons 7/8. RT-PCR was performed using SensiFAST Sybr Hi Rox Mix (Bioline). All RT-PCR reactions were performed using the 7900HT Fast Real-Time PCR System (Applied Biosystems). The experiments were carried out in triplicate for each data point. The housekeeping gene β-Actin was used as the internal control. Relative gene expression was calculated using the $2^{-\Delta CT}$ method, where the ΔCT was calculated using the differences in the mean CT between the selected genes and the internal control (β -Actin).

List of Primers:

BARD1β For: GCTGCTCGCGTTGATTTGAA

BARD1β Rev: ACGCTGCCCAGTGTTCATTAC

BARD1 FL For: ATGAAATCGCTATTGCTGCTACC

BARD1 FL Rev: ACGCTGCCCAGTGTTCATTAC

β-Actin For: CGTGCTGCTGACCGAGG

β-Actin Rev: GAAGGTCTCAAACATGATCTGGGT
